# A clean road to international trade: Environmental regulations and the cleanliness of export enterprises

**DOI:** 10.1016/j.heliyon.2023.e21180

**Published:** 2023-10-19

**Authors:** Weijian Du, Yuhuan Fan, Mengjie Li

**Affiliations:** a School of Economics, Shandong Technology and Business University, Yantai, Shandong, 264005, China; b School of Management and Economics, Beijing Institute of Technology, 100081, Beijing, China

**Keywords:** Environmental regulations, Export enterprises, Clean transformation, Extensive margin, Intensive margin

## Abstract

This study explores the impact of environmental regulations on the clean product exports of firms and the mechanism of this impact from the dual perspectives of the extensive and intensive margins. It uses matched micro data and aims to explore the clean road to international trade. According to the benchmark regression, environmental regulations improve the export intensity of clean products. However, their impact on the export probability of clean products is not significant. That is, environmental regulations promote the intensive margin of enterprise clean product exports but do not promote the extensive margin of enterprise clean product exports. The mechanism analysis shows that the cost effect is an important way for environmental regulations to promote the cleanliness of export products. However, the channel of technological innovation is not verified. In addition, compared with command-and-control and voluntary public policy tools, market-inspired environmental regulations have a stronger promoting effect on the cleanliness of export enterprises. This study provides microevidence and a policy basis for improving the environmental policy system and for the sustainable development of international trade.

## 1 Introduction

China's economic progress has been extraordinary in recent years. The rapid increase in export commerce has played a significant part in China's economic development. However, with the expansion of exports and economic aggregate growth, environmental pollution is becoming increasingly serious. To achieve sustainable economic development and reduce the negative impact of enterprises' production behavior on the natural environment, the Chinese government has continuously strengthened its control of enterprises' pollution emissions. China's pollution-intensive products, such as chemical, pulp and iron and steel products, account for a large share of the export market.

Although the relationship between environmental regulations and international trade has been extensively researched, past studies have mostly examined the trade effect of environmental regulations at the macro level, with minimal discussion of the dual margins and environmental policy tools. The objective of this research is to investigate the degree and mechanisms of the impact of environmental regulation intensity and different policy tools on the cleanliness of export firms from the dual margin perspective to explore the clean road to international trade. To achieve this objective, this work mainly includes the following content. First, the impacts of environmental regulations on whether firms export clean products and how many clean products firms export are analyzed. Second, this work explores the mechanism of the impact of environmental regulation on the cleanliness of export firms based on the channels of compliance costs and technological innovation. Finally, the heterogeneous effects of environmental regulations on the cleanliness of export firms under different types of policy tools are investigated. This work can help realize a win–win situation of environmental improvement and export trade transformation and promote sustainable economic development.

Compared to earlier research, this research makes the following contributions. In terms of the research perspective, this study investigates the impact of environmental policy on whether microenterprises export clean products and how many products they export, and it discusses the clean transformation of export enterprises from the dual perspectives of the extensive and intensive margins. In terms of research data, the empirical analysis of this study is based on matched data from the China Industrial Enterprise, China Enterprise Pollution Emission and China Customs Databases, providing a large sample dataset containing detailed information on enterprise production, pollution emissions and export products. The application of these data improves the representativeness and credibility of the research conclusions. In terms of the research framework, this study introduces the differential characteristics of environmental regulations and further explores how different types of environmental regulation tools affect the cleanliness of export enterprises.

The structure of this work is as follows. The second section is a review of the relevant research and proposes the theoretical hypotheses. The research design, which includes the model formulation, indicator development, and data sources, is presented in the third section. The fourth section conducts empirical analysis, including benchmark, robustness, and heterogeneity analyses on environmental regulations and the cleanliness of export firms. The fifth section is a continuation of the topic, including internal mechanisms, policy tools analysis and further discussion. The conclusion and policy implications are presented in the final section.

## 2 Research review and theoretical hypotheses

### 2.1 Research review

In recent years, a rising number of academics have focused on the link between environmental regulations and international trade. According to a review of available studies, the influence of environmental regulation on export trade is primarily separated into promotion and inhibition theories. The studies are summarized in Table 1. According to promotion theory, environmental regulations can support an improvement in manufacturing technology and increase international competitiveness, hence promoting export trade [[1], [2], [3], [4], [5], [6], [7], [8]]. According to inhibition theory, environmental regulations raise firm expenses and stifle the expansion of export trade [[9], [10], [11], [12], [13], [14], [15], [16]].

**Table 1:** Summary of past studies on environmental regulations and international trade.^1^

Author	Environmental policy variable	International trade variable	Outcomes
Chen et al. [1]	The removal rate of SO2 emissions	Export competitiveness	+
Cheng et al. [2]	Two control zones policy	Export decision	+
Lin and Linn [3]	European passenger vehicle greenhouse gas emission standards	Product attributes	+
Qiang et al. [4]	Environmental regulation intensity	Export volume	+
Sun et al. [5]	Cleaner production standards	Domestic value-added rate	+
Sun et al. [6]	Voluntary environmental regulations	Solar energy industry trade flows	+
Xu et al. [7]	Environmental regulation intensity	Global value chain	+
Yang et al. [8]	Occurrence frequency of environment-related words in municipal government work reports	Export technology structure	+
Cherniwchan and Najjar [9]	Air quality standards	Export decision	–
Kawabata and Takarada [10]	Environmental tax	Intermediate trade	–
Lee and Ho [11]	Environmental regulation stringency (0–6)	Export diversification	–
Shi and Xu [12]	Eleventh Five-Year Plan	Exports volume	–
Wu et al. [13]	Environmental regulation intensity	Export-oriented production	–
Xie and Zhang [14]	Environmental regulation intensity	FDI	–
Zhang et al. [15]	City Air Pollution Prevention and Control Program	Export volume	–
Zhang et al. [16]	Wastewater discharge standard	Export values	–

### 2.2 Theoretical hypotheses

Based on the existing theory of the pollution haven and Porter hypotheses, environmental regulations, as an important means of solving problems related to resource allocation and pollution, may affect enterprises’ exports through compliance costs and technological innovation effects [[17], [18], [19]]. From the perspective of the compliance cost effect, when environmental regulations are strengthened, export enterprises must pay more emission charges to environmental protection departments, which increases the production and operating costs of enterprises [20,21]. In addition, enterprises may increase their investment in environmental protection to meet stricter local environmental protection standards to promote enterprise cleanliness [22,23]. From the perspective of the technological innovation effect, when faced with strict environmental regulations, export enterprises may have a stronger motivation to carry out technological innovation and improve their production efficiency and production processes, thus realizing the clean transformation of export enterprises [[24], [25], [26]], compared to when faced with less strict environmental regulations. Therefore, Hypothesis 1 of this research is proposed as follows:

Hypothesis 1 Environmental regulations can promote the cleanliness of export enterprises through the effects of compliance costs and technological innovation.

According to research, different types of environmental regulations may differ in terms of their environmental protection purposes and government implementation modes [27]. The command-and-control environmental regulations used by the government to formulate mandatory regulations to limit pollution discharges achieve the goal of reducing the amount of pollution discharges by setting environmental standards or imposing administrative penalties on enterprises [28]. Market-inspired environmental regulations mainly guide enterprises to actively reduce pollution emissions through market forces [29]. Since the government does not directly intervene in the emission reduction behavior of enterprises, such behavior is more flexible. Voluntary public environmental regulations are a tool for enterprises to reduce emissions under the pressure of public supervision [30]. Command-and-control environmental regulations are both mandatory and timely, but they lack flexibility because they must be carried out by a certain time. Market-inspired environmental regulations give enterprises an increased right to independent choice and have a certain degree of compulsion and flexibility. The level of enforcement of voluntary public environmental regulations is determined by people's environmental awareness and the system for environmental reporting. Therefore, Hypothesis 2 of this study is proposed as follows:

Hypothesis 2 Command-and-control, market-inspired, and voluntary public environmental regulations have heterogeneous effects on the cleanliness of export enterprises.

## 3 Research design

### 3.1 Model specification

A microeconometric model is primarily concerned with the behavior of firms or people. The sample data are typically panel or cross-sectional data, with a large number of observations and substantial variability among the samples [31,32]. In this research, the data are characterized by micro firms, panel data, and big sample observations. As a result, a microeconometric model is utilized to examine the influence of environmental regulations on export firms’ cleanliness. The benchmark model employs a two-way fixed effects model to eliminate the interference of unobservable factors, introducing individual fixed effects to control for the interference of factors that do not change over time and time fixed effects to control for the interference of factors that do not change with individual characteristics. The employment of a microeconometric model aids in lowering estimation bias and in increasing the trustworthiness of research outcomes^2^. The benchmark regression formula is shown in formula (1).

(1) $$Cexoport_{it}=\alpha +\beta \;\ln\;ERI_{it}+\gamma X_{it}+v_{i}+v_{t}+\varepsilon_{it}$$

where *i* represents the enterprise and *t* represents the year. *Cexport*_*it*_ represents the cleanliness of export enterprises, which is mainly related to the extensive and intensive margins of clean product exports. *ERI*_*it*_ represents the intensity of environmental regulations at the enterprise level. *X*_*it*_ is the control variables. *v*_*i*_ and *v*_*t*_ represent individual and year fixed effects, respectively. *ε*_*it*_ represents the random disturbance term.

### 3.2 Indicator construction

**Cleanliness of export enterprises *Cexport***_***it***_**.** The extensive margin of clean product exports is measured by whether the clean products of enterprise *i* are exported in year *t*. The intensive margin of clean product exports is measured by the export intensity of the clean products of enterprise *i* in year *t*. Matching with the *HS* code of clean products is carried out based on the *HS* code of the enterprise export product information in the China Customs Database.

**Environmental regulation intensity *ERI***_***it***_**.** When measuring environmental regulations at the enterprise level, the method of matching enterprise variables with industry or regional environmental regulations may not accurately reflect the actual environmental regulation intensity faced by enterprises. Regarding the actual situation of Chinese enterprise pollution emissions, using the chemical oxygen demand (*COD*) removal rate as a form of environmental regulation can help enterprises avoid the impact of pollutants discharged by a few industries, mainly large state-owned enterprises, on the results [33,34]. Regarding data availability, the China Pollution Emission Database provides more detailed information on the *COD* production and emissions of enterprises than other pollution emission data, which can maximize the analysis sample size and representativeness of the estimated results. Therefore, the *COD* removal rate is used in this research to measure enterprise environmental regulation intensity.

**Control variables *X***_***it***_**.** The control variables include capital intensity, the business lifespan, enterprise scale and enterprise productivity. The ratio of fixed capital to the number of workers is used to calculate capital intensity (*CI*). The difference between the current year and the year of establishment of the enterprise plus 1 is used to calculate the business lifespan (*Age*). The fixed capital of the enterprise is used to calculate enterprise scale (*Scale*). The ratio of total output to the number of workers is used to calculate enterprise productivity (*LP*).

### 3.3 Data sources

This study uses 2000–2010 microlevel data from the China Industrial Enterprise, China Enterprise Pollution Emission, and China Customs Databases.^3^ The National Bureau of Statistics' China Industrial Enterprises Database provides yearly statistics. These statistics mostly consist of quarterly and yearly reports provided to the local statistical office by the sample firms. All state- and non-state-owned industrial firms over a certain size are included in the sample. Enterprise information comprises an enterprise's fundamental condition and financial data. Samples with statistical flaws in industrial firm data are deleted and categorized in accordance with fundamental accounting principles. The China Enterprise Pollution Emission Database combines yearly data from China's Ministry of Ecology and Environment with a quarterly survey of significant polluting firms. Major polluters are those whose pollution emissions account for more than 85 % of the total emissions in each county. The statistical elements primarily comprise the names of firms, their locations, and significant pollution emissions. The Chinese Customs Database publishes monthly data collected by Chinese Customs that cover all transactions entering and exiting the customs area. Basic information (e.g., firm name, ownership, and address), commodity information (e.g., variety, price, and quantity), the destination market, the transit mode, and the trading mode are examples of statistical items. The China Customs Database offers monthly data on import and export transaction records, and for this reason, enterprise export data are aggregated into yearly statistics based on the destination country and product HS4 code.

The matching process for the data is as follows. First, the data from the China Industrial Enterprise Database are processed based on Brandt, Van Biesebroeck [35], and the panel data of industrial enterprises are formed. Second, the data from the China Enterprise Pollution Emission Database are processed by a similar method to form pollution panel data. Third, based on the enterprise name and unique identification code, industrial enterprise-pollution panel data are created by combining the China Industrial Enterprise and China Enterprise Pollution Emission Databases. Finally, based on Upward, Wang [36], the China Customs Database is merged with the industrial enterprise-pollution panel data.

In China, merging methodologies are commonly employed in research on microenterprises. However, due to the limits of the various databases, certain sample selection biases are seen. The combined samples may be biased toward large-scale, polluting industries. The China Industrial Enterprise Database contains only samples of firms larger than a specified size, resulting in some information loss in the combined data of small and medium-sized enterprises. The China Enterprise Pollution Emission Database contains only samples of significant polluting firms, resulting in some information loss of clean enterprises' integrated data. As a result, the combined dataset includes only a subset of the three large datasets. Nonetheless, the combined data remain the largest microsample dataset available for studying Chinese firms' exports and cleanliness transition. These large-sample micro data have universal representative significance and can provide strong data support for the empirical analysis of this research.

## 4 Empirical analysis

### 4.1 Benchmark analysis on environmental regulations and the cleanliness of export firms

The panel two-way fixed effects model is used to examine how environmental regulations affect the cleanliness of export firms. Table 2 shows the results of the impact of environmental regulations on the extensive margin of clean product exports. As shown in Table 2, the coefficients of *ERI* are not significant, which indicates that environmental regulations cannot increase the export probability of enterprises' clean products. One possible explanation for this result is that there are high fixed costs in the export of different enterprise products and that the adjustment of production equipment brought by product conversion affects enterprises' export product portfolio. For enterprises, when the cost increase caused by environmental regulations is less than that caused by product conversion, environmental regulations do not encourage enterprises that export pollution-intensive products to export cleaner products.

**Table 2:** Impact of environmental regulations on the extensive margin of clean product exports.

	Extensive margin of clean product exports				
	(1)	(2)	(3)	(4)	(5)
*ERI*	0.0022 (0.0077)	0.0027 (0.0082)	0.0027 (0.0082)	0.0024 (0.0082)	0.0020 (0.0082)
*CI*		−0.0061* (0.0032)	−0.0061* (0.0032)	−0.0186*** (0.0049)	−0.0243*** (0.0053)
*Age*			0.0028 (0.0067)	0.0018 (0.0068)	0.0021 (0.0068)
*Scale*				0.0122*** (0.0036)	0.0149*** (0.0037)
*LP*					0.0117*** (0.0043)
*Constant*	0.2137*** (0.0054)	0.2424*** (0.0154)	0.2360*** (0.0218)	0.1656*** (0.0300)	0.0992** (0.0388)
*Time fixed effect*	Yes	Yes	Yes	Yes	Yes
*Individual fixed effect*	Yes	Yes	Yes	Yes	Yes
*Observations*	34,755	31,107	31,094	31,094	31,094

Note that *, **, and *** denote significance at the 10 %, 5 %, and 1 % levels, respectively. Standard errors are shown in parentheses.

The influence of the control variables on the extensive margin of clean product exports is as expected. The capital intensity variable is negative and significant, indicating that as the capital intensity of enterprises increases, the export probability of clean products decreases. Enterprises with high capital intensity tend to be heavy industrial enterprises and are thus more likely to export pollution-intensive products. The enterprise scale variable is positive and significant, indicating that with the expansion of enterprise scale, the export probability of clean products increases, and larger enterprises are more likely than smaller enterprises to diversify the types of products that they export. The enterprise productivity variable is positive and significant, indicating that with the increase in enterprise productivity, the export probability of clean products increases. Enterprises with high productivity tend to have technological advantages and are more inclined to increase the number of export product categories.

The impact of environmental regulations on the intensive margin of clean product exports is further investigated. The results of which are shown in Table 3. The coefficients of *ERI* are all significantly positive, indicating that environmental regulations improve the export intensity of enterprises' clean products and promote the cleanliness of export enterprises from the intensive margin. One possible explanation for this result is that environmental regulations may reduce the opportunity cost of exporting clean products to a certain extent so that enterprises are more inclined to expand their production scale of clean products and ultimately increase the proportion of exports of such products.

**Table 3:** Impact of environmental regulations on the intensive margin of clean product exports.

	Intensive margin of clean product exports				
	(1)	(2)	(3)	(4)	(5)
*ERI*	0.0071*** (0.0025)	0.0061** (0.0027)	0.0062** (0.0027)	0.0061** (0.0027)	0.0060** (0.0027)
*CI*		−0.0017 (0.0011)	−0.0017 (0.0011)	−0.0045*** (0.0016)	−0.0062*** (0.0017)
*Age*			0.0025 (0.0022)	0.0022 (0.0022)	0.0024 (0.0022)
*Scale*				0.0028** (0.0012)	0.0036*** (0.0012)
*LP*					0.0034** (0.0014)
*Constant*	0.0898*** (0.0018)	0.0981*** (0.0050)	0.0925*** (0.0072)	0.0764*** (0.0098)	0.0573*** (0.0127)
*Time fixed effect*	Yes	Yes	Yes	Yes	Yes
*Individual fixed effect*	Yes	Yes	Yes	Yes	Yes
*Observations*	34,755	31,107	31,094	31,094	31,094

### 4.2 Robustness analysis of environmental regulations and the cleanliness of export firms

To test the robustness of the explanatory variable, this research measures the intensity of environmental regulations using the investment in industrial pollution control per unit of industrial output value. Columns (1) and (2) in Table 4 analyze the impact of environmental regulations on the cleanliness of enterprises' export products from the dual perspectives of the extensive and intensive margins. Columns (3) and (4) further introduce the industry and region variables, which control for the interference of region and industry factors, for robustness analysis. In addition, provincial capital cities, as political and administrative centers within regions, often have differentiated environmental regulatory policies compared to general regions. To overcome the potential biased impact of this feature, this research further excludes samples from municipalities directly under the central government, provincial capitals, and subprovincial cities. The results in Table 4 show that environmental regulations significantly increase the export intensity of enterprises’ clean products but do not increase the export probability of clean products, confirming the conclusions of the benchmark regression.

**Table 4:** Robustness test I: Replacement of the explanatory variable and sample adjustments.

	Explanatory variable replacement		Industry and region factors		Excluding provincial capital cities	
	(1) *Extensive*	(2) *Intensive*	(3) *Extensive*	(4) *Intensive*	(5) *Extensive*	(6) *Intensive*
*ERI*	2.6326 (2.0316)	1.6412** (0.6874)	0.0018 (0.0082)	0.0057** (0.0027)	−0.0030 (0.0099)	0.0055* (0.0033)
*Constant*	−0.0420 (0.0443)	0.0082 (0.0150)	0.0413 (0.2850)	0.0311 (0.0935)	0.0570 (0.0598)	0.0692*** (0.0197)
*Control variables*	Controlled	*Controlled*	*Controlled*	*Controlled*	*Controlled*	*Controlled*
*Time fixed effect*	Yes	Yes	Yes	Yes	Yes	Yes
*Individual fixed effect*	Yes	Yes	Yes	Yes	Yes	Yes
*Industry factors*	No	No	Yes	Yes	No	No
*Region factors*	No	No	Yes	Yes	No	No
*Observations*	37,172	37,172	31,094	31,094	18,574	18,574

The People's Republic of China's 11th Five-Year Plan's Outline for National Economic and Social Development officially included environmental target constraints in its official assessment targets for the first time. Will this constraint policy interfere with enterprise exports? To exclude the interference of policy shocks, the samples are divided into two stages—before and after policy implementation—for subsample regression, as shown in Table 5. Before policy implementation, environmental regulations do not significantly increase the export probability and proportion of clean products. When environmental targets are formally included in official assessments, environmental regulations still have no significant impact on whether enterprises export clean products, but they significantly increase the export intensity of clean products. This result may be explained by the fact that the inclusion of environmental targets in official assessments improves the enforcement of environmental regulations and reduces the opportunity cost of clean product exports, leading enterprises to be more inclined to export clean products.

**Table 5:** Robustness test II: Exclusion of policy interference.

	Before policy implementation		After policy implementation	
	(1) *Extensive*	(2) *Intensive*	(3) *Extensive*	(4) *Intensive*
*ERI*	0.0045 (0.0118)	0.0054 (0.0038)	−0.0032 (0.0145)	0.0096* (0.0049)
*Constant*	0.1896*** (0.0521)	0.0588*** (0.0169)	0.0615 (0.0923)	0.0761** (0.0311)
*Control variables*	Controlled	Controlled	Controlled	Controlled
*Time fixed effect*	Yes	Yes	Yes	Yes
*Individual fixed effect*	Yes	Yes	Yes	Yes
*Observations*	19,508	19,508	11,586	11,586

### 4.3 Heterogeneity analysis of environmental regulations and the cleanliness of export firms

In the face of environmental regulations, enterprises with different capital characteristics may have significant differences in terms of clean product exports. Table 6 further investigates how capital characteristics affect the association between environmental regulations and clean product exports. The results show that environmental regulations can increase the export intensive margin of clean products in capital-intensive enterprises but cannot increase the export extensive margin of clean products in these enterprises. For labor-intensive enterprises, the extensive margin and intensive margin of clean product exports are not significantly impacted by environmental regulations. The p value of the coefficient difference test is significant at the 10 % level, which proves that the coefficient difference between groups is significant^4^. One possible explanation for this result is as follows. Compared with capital-intensive enterprises, labor-intensive enterprises export relatively clean products, and environmental regulations do not significantly affect the emission reduction costs of labor-intensive enterprises. Therefore, among these enterprises, environmental regulations have a stronger role in promoting the cleanliness of capital-intensive enterprise exports.

**Table 6:** Heterogeneity analysis I: Introducing capital characteristics.

	Extensive margin		Intensive margin	
	(1) Labor-intensive	(2) Capital-intensive	(3) Labor-intensive	(4) Capital-intensive
*ERI*	−0.0115 (0.0117)	0.0031 (0.0208)	0.0034 (0.0039)	0.0141** (0.0071)
*Constant*	0.0558 (0.0691)	0.0259 (0.1765)	0.0784*** (0.0227)	−0.0758 (0.0601)
*Control variables*	*Controlled*	*Controlled*	*Controlled*	*Controlled*
*Time fixed effect*	Yes	Yes	Yes	Yes
*Individual fixed effect*	Yes	Yes	Yes	Yes
*Observations*	14,771	3803	14,771	3803
*Coefficient difference p value*	0.047		0.098	

In addition, the distinctive feature of Chinese enterprises is the coexistence of various types of ownership, and the operating background and business environment of different types of ownership are clearly different. The results in Table 7 regarding the effects of environmental regulations on the cleanliness of export firms with various ownership structures reveal that environmental regulations can cause non-stated-owned enterprises to increase their export intensive margin of clean products but cannot cause state-owned enterprises to increase their export intensive margin of clean products. For state-owned and non-stated-owned enterprises, environmental regulations do not significantly increase the export extensive margin of clean products. The p value of the coefficient difference test is significant at the 10 % level, which proves that the coefficient difference between groups is significant. One possible explanation for this result is that the business performance and employment guarantee of state-owned enterprises have a significant part in the promotion and assessment of local officials, which may lead to the incomplete enforcement of environmental regulations among state-owned enterprises.

**Table 7:** Heterogeneity analysis II: Introducing ownership characteristics.

	Extensive margin		Intensive margin	
	(1) state-owned	(2) non-state-owned	(3) state-owned	(4) non-state-owned
*ERI*	−0.0031 (0.0262)	0.0028 (0.0086)	0.0045 (0.0104)	0.0071*** (0.0025)
*Constant*	−0.1911 (0.1433)	0.0438 (0.0532)	−0.1332** (0.0567)	0.0652*** (0.0157)
*Control variables*	Controlled	Controlled	*Controlled*	*Controlled*
*Time fixed effect*	Yes	Yes	Yes	Yes
*Individual fixed effect*	Yes	Yes	Yes	Yes
*Observations*	6205	24,889	6205	24,889
*Coefficient difference p value*	0.099		0.099	

## 5 Expansion analysis

### 5.1 The internal mechanisms of the impact of environmental regulations on the cleanliness of export firms

This study confirms that environmental regulations might, to a certain extent, encourage the cleanliness of export operations. To verify the hypothesis, this section uses an interaction effect model to discuss the mechanisms through which environmental regulations promote export enterprises’ clean transformation through the effects of compliance costs and technological innovation. The compliance cost effect is measured by enterprise production costs. The technological innovation effect is measured by enterprise patent applications.

The mechanism of the compliance cost effect is described in Columns (1) and (2) in Table 8. Column (2) shows that the interaction coefficient between environmental regulations and compliance costs is significantly positive, indicating that environmental regulations increase the intensive margin of clean product exports by increasing enterprises’ production costs. The interaction coefficient between environmental regulations and compliance costs in Column (1) is not significant, indicating that environmental regulations do not have a significant impact on the extensive margin of clean product exports for enterprises.

**Table 8:** Internal mechanism analysis.

	Compliance cost effect		Technological innovation effect	
	(1) *Extensive margin*	(2) *Intensive margin*	(3) *Extensive margin*	(4) *Intensive margin*
*ERI*	0.0032	0.0064**	0.0018	0.0059**
	(0.0082)	(0.0027)	(0.0082)	(0.0027)
*Cost*	−0.0093	−0.0377***		
	(0.0376)	(0.0123)		
ERI*Cost	0.2947***	0.0994***		
	(0.0936)	(0.0306)		
*Patent*			0.0075*	−0.0005
			(0.0039)	(0.0013)
ERI*Patent			0.0170	−0.0002
			(0.0112)	(0.0037)
*Constant*	0.0274	0.0645***	−0.0057	0.0193
	(0.0539)	(0.0176)	(0.0495)	(0.0163)
*Control variables*	Controlled	Controlled	Controlled	Controlled
*Time fixed effect*	YES	YES	YES	YES
*Individual fixed effect*	YES	YES	YES	YES
*Observations*	31,070	31,070	31,094	31,094

Columns (3) and (4) in Table 8 detail the mechanism underlying the impact of technological innovation. Columns (3) and (4) show that the interaction coefficients between environmental regulations and technological innovation are not significant. These results do not support the notion that the technological innovation effect of enterprises in the sample interval is the transmission channel through which environmental regulations promote the cleanliness of export enterprises. The conclusions are consistent with those of existing research [37,38]. Enterprises need a large amount of capital investment in the early stage of technological innovation and face the risk of innovation failure. Thus, environmental regulations do not necessarily lead to the technological innovation of enterprises.

### 5.2 The heterogeneous impacts of environmental regulation tools on the cleanliness of export firms

To verify *Hypothesis* 2, this section analyzes the heterogeneous impact of different environmental regulatory policy tools on the cleanliness of export enterprises. First, the impact of command-and-control environmental regulations on the cleanliness of export enterprises is investigated. At present, the “three simultaneities” system can well represent the application of command-and-control environmental regulations in China. Other systems are relatively short in terms of period, and their scope is relatively restricted. Therefore, the ratio of the “three simultaneities” system of environmental investment to industrial value added is used to measure command-and-control environmental regulations. The findings of the regressions, which are displayed in Column (1) and (2) in Table 9, demonstrate that command-and-control environmental regulations are ineffective in significantly promoting the clean transformation of export firms. This result may be explained by the fact that command-and-control environmental policies primarily influence the pollution emissions of firms by establishing obligatory emission limits and technical requirements. This aspect makes these policies unable to effectively incentivize the clean transformation of export businesses.

**Table 9:** Impact of heterogeneous environmental regulations on the cleanliness of enterprise exports.

	Command-and-control		Market-inspired		Voluntary public	
	(1) *Extensive*	(2) *Intensive*	(3) *Extensive*	(4) *Intensive*	(5) *Extensive*	(6) *Intensive*
*ERI*	0.0300	−0.1447	2.2484	0.9578*	0.0038	0.0015
	(0.2599)	(0.0946)	(1.4778)	(0.5375)	(0.0032)	(0.0012)
*Constant*	0.0559**	0.0610***	0.0470*	0.0565***	0.0515**	0.0586***
	(0.0238)	(0.0087)	(0.0245)	(0.0089)	(0.0241)	(0.0088)
*Control variables*	Controlled	Controlled	Controlled	Controlled	Controlled	Controlled
*Time fixed effect*	YES	YES	YES	YES	YES	YES
*Individual fixed effect*	YES	YES	YES	YES	YES	YES
*Observations*	65,551	65,551	65,577	65,577	65,577	65,577

The impacts of market-inspired environmental regulations on the cleanliness of export firms are examined. One typical market-inspired environmental tool is the sewage charge system in China. Therefore, this study measures market-inspired environmental regulations by the ratio of sewage fees to pollution emissions. The regression results, which are presented in Columns (3) and (4) in Table 9, show that market-inspired environmental regulations increase the export intensity of clean products but do not improve the export probability of clean products. One possible explanation for this result is that market-inspired environmental regulations require enterprises to pay fees based on environmental standards to internalize the external costs brought by their pollution behaviors. This aspect provides strong flexibility and incentives.

Finally, the impact of voluntary public environmental regulations on the cleanliness of enterprise exports is investigated. Referring to Du et al. [27], voluntary public environmental regulations are calculated by the ratio of environmental complaints in each province to the regional population. Columns (5) and (6) in Table 9 present the regression results, demonstrating that voluntary public environmental regulations cannot improve the cleanliness of enterprise exports. One possible explanation for this result is that such regulations do not have a mandatory binding effect, and their implementation intensity depends on the public's environmental awareness and the corresponding environmental protection reporting mechanism. At present, China's environmental education level is still not high, and the public's awareness of ecological environmental protection is insufficient.

### 5.3 Further discussion^5^

With the emergence of pollution problems and the deepening of economic globalization, the interaction between environmental policies and international trade has attracted increasing attention [1,39]. Theoretical research on the influence of environmental regulations on firm exports includes mainly the pollution haven hypothesis [3,40,41] and the Porter hypothesis [5,42,43]. Research on pollution havens hypothesizes that environmental regulations may lead to an increase in environmental costs [9,44], which in turn can lead to a reallocation of the resources of enterprises. Research on the Porter hypothesis holds that the pressure of environmental regulations may stimulate the technological innovation of enterprises [8,45,46], which in turn offsets the cost increase caused by environmental regulations. This research investigates the impact of environmental policy on whether microenterprises export clean products and how much they export, and it discusses the clean transformation of export enterprises from the dual perspectives of the extensive and intensive margins. The results show that environmental regulations promote the intensive margin of enterprise clean product exports, and production costs and investment in emission reduction equipment are important channels. The results support the pollution haven hypothesis, i.e., that environmental regulations lead to a reallocation of the resources of export enterprises [1,12,47]. This research has important theoretical and practical significance for promoting the coordinated development of China's environmental improvement and economic growth.

Additionally, this research introduces the differential characteristics of environmental regulations and further explores how different types of environmental regulation tools affect the cleanliness of export enterprises. The results show that compared with command-and-control and voluntary public policy tools, market-inspired environmental regulations have a stronger promoting effect on the cleanliness of export enterprises. The results are consistent with those of existing research [[48], [49], [50]], demonstrating that market-inspired environmental regulations are more effective because of the strong flexibility and incentives [49,51]. This research provides a scientific basis and microempirical evidence for the selection of environmental policy tools.

## 6 Conclusions and policy implications

With the increased breadth and length of haze in China, environmental preservation has become a top priority for citizens and policymakers alike. Environmental regulation policies are direct and effective methods of pollution management and environmental protection. Only a few studies have examined whether China's environmental rules impede economic development, particularly the expansion of export trade. Research on this topic is beneficial for improving the environmental policy framework and supporting the clean transformation of China's international trade. This research has significant theoretical and practical implications for advancing China's integrated development of environmental improvement and economic growth.

This study presents an examination of the influence of environmental regulations on the cleanliness of enterprise exports from the dual perspectives of the extensive and intensive margins based on the China Industrial Enterprise, China Enterprise Pollution Emission, and China Customs Databases, and it reaches the following conclusions. First, environmental regulations can increase the intensive margin of clean product exports but cannot increase the extensive margin. Second, compared with other enterprises, environmental regulations play a stronger role in promoting the clean export transformation of capital-intensive and non-state-owned enterprises. Third, the compliance cost effect is an important mechanism through which environmental regulations can promote the cleanliness of enterprise exports, while as a mechanism, the technological innovation effect is not verified. Finally, market-inspired environmental policies have the strongest promoting effect on the clean transformation of enterprise exports in the sample interval, while the results of command-and-control and voluntary public environmental measures are found to be relatively weak.

On the basis of the study findings, the following policy implications are noted. First, the government should gradually improve and develop the environmental regulation system to force the clean transformation and upgrading of China's foreign trade enterprises. The government should take advantage of the way in which environmental regulations advance the export extensive margin of clean products, guide some pollution-intensive product export enterprises to export clean products through environmental regulations, and realize the transformation of the export growth of China's enterprises' clean products under environmental regulation constraints from the intensive margin to both the intensive margin and the extensive margin. Second, the government must develop distinct environmental regulation rules based on various firm characteristics. At present, the economic development level and industry characteristics of different regions in China are quite different. Policymakers should combine the actual situations of different regions, industries and enterprises with different ownership structures, implement special governance and key inspections, improve the punishment of enterprises' illegal emissions, and achieve a synergy between the pollution reduction and trade development of different export enterprises. Third, government departments should provide policy support to enterprises to guide them in achieving a clean transformation through technological innovation under environmental regulation constraints. Regarding the impact of environmental policy, some enterprises may directly introduce emission reduction equipment rather than engaging in technological innovation to achieve environmental standards. Therefore, while strengthening environmental regulations, government departments can appropriately provide enterprises with preferential support policies such as R&D subsidies and innovation tax credits to allow them to better take advantage of the role of technological innovation in the green development of international trade. Finally, the government should improve the market mechanism and supervision mechanism and fully exploit the guiding role of market-inspired environmental policies and the supervisory role of voluntary public environmental regulations. The government should establish a pollution charge system with the environmental protection tax system as the core, further improve the national emission rights trading market, and accelerate the interaction and coordination between the environmental protection tax and emission rights trading system. Additionally, the enterprise environmental information disclosure mechanism and people's awareness of environmental pollution supervision should be improved, and the role of voluntary public environmental regulations should be gradually expanded.

This research also has some limitations that warrant further attention in future work. The internal mechanisms of the effect of environmental regulations on the cleanliness of export firms are complex and diverse. This research uses an interaction effect model to analyze the mechanisms from the perspectives of the effects of compliance costs and technological innovation. There may be mechanism recognition bias, and other potential mechanisms may be ignored to some extent. Therefore, how to adopt more accurate mechanism identification methods and test more potential mechanisms will become the direction of further research. Furthermore, additional work is necessary due to the sample data limitations in this research. Because of a lack of crucial indicators and the poor quality of the follow-up period, this research included only data from 2000 to 2010. As a result, the conclusions of this research are constrained by the sample period. Future work can update the sample period in China through on-the-ground investigations and questionnaire surveys, broadening this research and allowing broader conclusions to be drawn.

## Author contribution statement

Weijian Du: conceived and designed the experiments; contributed reagents, materials, analysis tools or data. Yuhuan Fan: wrote the paper; analyzed and interpreted the data. Mengjie Li: performed the experiments.

## Funding statement

This research was sponsored by the 10.13039/501100001809National Natural Science Foundation of China (72274112, 71803102), Taishan Scholars Project Funding (tsqn202211237, tsqn202306273), the 10.13039/501100007129Shandong Provincial Natural Science Foundation (ZR2020QG041) and the 10.13039/501100002858China Postdoctoral Science Foundation (2021M690018).

## Data availability statement

The data presented in this study are available upon request from the corresponding author.

## Additional information

No additional information is available for this study.

## Declaration of competing interest

The authors declare that they have no known competing financial interests or personal relationships that could have appeared to influence the work reported in this paper.

## Footnotes

1 The authors would like to thank the reviewers for the suggestion to present the main research in a table.

## Footnotes

2 The authors would like to thank the reviewers for the suggestion to add an introduction to the microeconometric model.

3 This sample period was selected primarily based on two factors: (1) the availability of key indicators and the caliber of the data samples; and (2) the lack of any significant changes in China's environmental policy and export market since 2010 that might have affected the reliability of the findings of this research.

4 The authors would like to thank a reviewer for the suggestion to test the intergroup coefficient differences.

5 The authors would like to thank the reviewers for their suggestion to add this discussion section.
